# Pitfalls in Tick and Tick-Borne Pathogens Research, Some Recommendations and a Call for Data Sharing

**DOI:** 10.3390/pathogens10060712

**Published:** 2021-06-07

**Authors:** Agustín Estrada-Peña, Aitor Cevidanes, Hein Sprong, Javier Millán

**Affiliations:** 1 Department of Animal Health. Faculty of Veterinary Medicine. University of Zaragoza, 50013 Zaragoza, Spain; 2 Research Group in Emerging Zoonoses, Instituto Agroalimentario de Aragón-IA2 (Universidad de Zaragoza-CITA), 50013 Zaragoza, Spain; syngamustrachea@hotmail.com; 3 Department of Animal Health, NEIKER-Basque Institute for Agricultural Research and Development, Basque Research and Technology Alliance (BRTA), Parque Científico y Tecnológico de Bizkaia P812, 48160 Derio, Spain; aitorcevi@gmail.com; 4 Center for Infectious Disease Control, National Institute for Public Health and the Environment, Mailbox 63, Room V353, Antonie van Leeuwenhoeklaan 9, P.O. Box 1, 3720 BA Bilthoven, The Netherlands; Hein.Sprong@rivm.nl; 5 Fundación ARAID, 50018 Zaragoza, Spain; 6 Facultad de Ciencias de la Vida, Universidad Andrés Bello, Santiago 8370146, Chile

**Keywords:** ticks, tick-transmitted pathogens, tick-surveys, prevalence reporting, GenBank, guidelines

## Abstract

An understanding of the relationships of ticks and tick-borne pathogens can only be achieved by integrating data from multiple studies. The publication of raw material is a necessary step for wide-area meta-analyses and study design, data collection and reporting require harmonization. This is an opinion paper, not a consensus position, and is open to debate. This work reflects our view about how data should be communicated in mainstream journals. We indicate rules that should be observed in recording weather data, to avoid serendipitous correlations between the density of ticks and climate variables and recommend the inclusion of raw data in reports. We stress the need for standardized methods to collect ticks that cannot be obtained by standard flagging. The reporting of infection rates of pathogens in ticks should avoid conclusions based on pure molecular findings in feeding ticks. Studies demonstrating the vectorial capacity of ticks should not be supported only by molecular surveys of feeding ticks. Vacuous conclusions about vectorial or reservoir status based solely on the finding of genomic material of a pathogen should be discouraged. We stress that phylogenetic studies based on random selection of sequences from GenBank are unsuitable. We firmly support the development of a dedicated server of curated sequences of ticks and pathogens as a standard for future studies.

## 1. Introduction

There is an increased awareness about tick-transmitted pathogens, in both human and animal populations. In recent years, dozens of studies have reported local, regional or even national infection rates of pathogens in ticks. This is a necessary step prior to issuing, for example, guidance on self-protection measures in humans or control campaigns in pets or livestock. Some countries are relatively well surveyed, while large areas of other countries are still poorly covered (compare, for example, the tick distribution maps for Europe and northern Africa) [1]. The last few decades have been a boiling pot for research on ticks and tick-transmitted pathogens. An increased recognition of the importance of these arthropods in human health is behind many of the initiatives and current international projects on the topic. We are entering into a new era of progress on the topic, in which the importance of ecological concepts, the relationships between wild and domestic animals, the effects of global change on local disease risk (and vice versa) and human habits are being re-considered. State-of-the-art molecular methods or the “big data” universe have been incorporated into the arsenal of techniques used by researchers studying ticks. However, along with new technologies and opportunities, pitfalls arise.

We must aim for the inter-comparability of data. We cannot design large enough studies to grasp the complexity of ticks and transmitted pathogens. Rather, we need to be able to reuse available data for meta-analyses. Some data might not be of direct relevance for a particular study but could be extremely useful for data integration into the larger picture. In the era of big data, we need to build a common and solid framework of harmonized protocols, interpretation of results and data presentation, over which hypotheses can be developed. Single studies can be considered pieces of a puzzle that should fit together to eventually illuminate the whole picture. Laboratory studies may clarify some issues, but in the field, communities of vertebrates and ticks exhibit complex dynamics involving vertebrates, vectors and pathogens. As researchers in the field, the co-authors realized that there is a lack of homogeneity in the way some studies are conducted, which impairs comparisons among the reported results. A prerequisite for data integration is that datasets are compatible and have some coherent and harmonized way of collection and storage. We realized that to move forward, it is necessary to: (i) achieve a minimum level of harmonization in the methodology, (ii) report data in a homogeneous format and (iii) remove from reports concepts that have already been rejected by science but that continue to plague publications.

This manuscript intends to promote the proper design and interpretation of tick-derived studies, harmonizing the procedures to collect and analyze information. If these methods do not yet exist, or a better approach is foreseen, we propose an agenda for future research and areas that should be developed. We will examine some basic questions of tick ecology and physiology, plus the circulation of pathogens, a concept commonly overlooked in the current research on the topic. This paper is also a plea to the research community to increase the availability of integrated raw data for future research about ticks, hosts and pathogens. This availability should be a requirement for publication of research on the topic because without such information, researchers cannot understand the real meaning of host-tick-pathogen relationships. Such an understanding is necessary to interpret the dynamics of these systems, to model the dynamics of a pathogen’s circulation, and to better understand the impact of the many environmental, landscape and human-derived factors on these complex systems. We do not intend to cover all the facets related to research on ticks, and for which a lack of harmonization has already been recognized.

## 2. Surveying Off-Host Ticks

Perhaps one of the first steps in any study regarding tick-borne pathogens is the collection of questing ticks. See Appendix A for definitions of common terms. The results on the density of questing ticks are commonly published together with a statistical analysis of the environmental variables that could explain the field findings in the short and/or the long term (i.e., variables operating at the time of collection or variables explaining the physiological age of the ticks). Researchers aim to compare tick density from different sites or periods of the year, or to establish where and when ticks became more prevalent. Harmonized protocols of collections are a must, aimed at the comparability of different surveys, even in distant areas (see below under point 2.2).

Probably the first issue with this lack of harmonization appears when researchers aim to correlate tick density with environmental features. If researchers want to compare published tick activity cycles at a large scale (i.e., continental) as part of a meta-analysis, the result will most probably suffer from lack of harmonization regarding the variables explaining seasonality, how these variables were recorded and how statistical associations were demonstrated. We propose here (i) the development of standardized collection protocols for use throughout a continent (i.e., cloth-dragging, but see [2] for gaps about the methods), (ii) the collection of a given stage and species at the same time by different teams and under different ecological conditions, (iii) a wide agreement among researchers on the explanatory variables to be used for correlation with tick abundance, (iv) agreement and use of standardized statistical methods for correlating tick density and weather/landscape variables and (v) the importance of long-term (several years) tick surveys conducted at the same site. The last point is of special importance since it could allow the detection of variations in tick “abundance” driven by abiotic (weather) and biotic (hosts) variables.

### 2.1. How Ticks Respond to Environmental Variables

**Figure 1:**
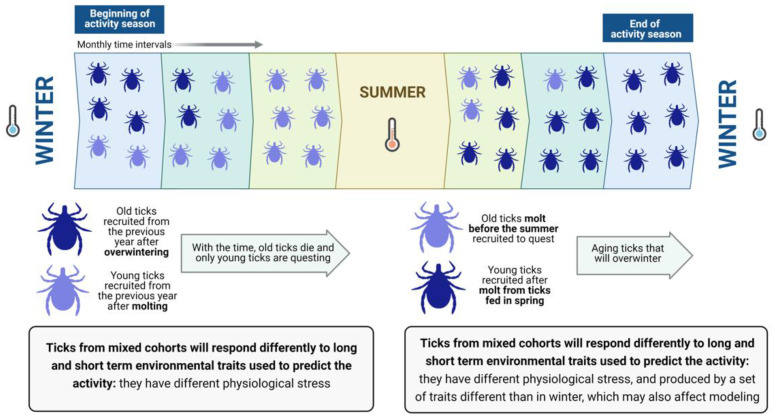
The coexistence of different tick cohorts in monthly samples may result in a different response to weather variables. The figure shows a hypothetical pattern of tick activity, in which they may not be active in summer because of high evaporation, and in winter, because of low temperature or short photoperiod. “Monthly time intervals” refer to an undetermined period of time. Even though the example is hypothetical, it is inspired by the realized activity of *Ixodes* spp. in parts of the Holarctic region. Ticks, thus, have different patterns of recruitment. In spring, newly molted ticks after winter (light blue) mix with ticks that passed the winter in quiescence and regained activity (dark blue). As spring moves into summer, more newly molt ticks incorporate into the questing stage, and old ticks from the previous year die or find a host. After the summer, ticks molt in spring (light blue) regain activity and young ticks (dark blue) result from molts. At the end of the autumn, ticks questing since spring have already most likely died or found a host, and most ticks may come from the molt after summer. Therefore, ticks of different physiological ages coexist in the same month and are collected together. This affects the modeling of the “response” of ticks to weather and enhances the need to provide raw data for weather with every report to allow reliable future meta-analyses. Created with BioRender.com.

Two or more cohorts of ticks may co-exist in the field [3] but this is not a rule for every species of tick. Ticks of similar physiological age may occur in sites with a strict weather seasonality that “resets” tick metabolic activity and separates the different periods of molting and activity. However, for populations of ticks that show long-lasting activity (i.e., continuous vitality throughout one complete year), cohorts of ticks of different physiological ages could coexist and quest together [4]. Therefore, “old” and “young” ticks overlap in space and time (Figure 1).

A pivotal problem is that researchers do not know the physiological age of the ticks they collected. The physiological age derives from the stress produced by past weather conditions (i.e., loss of water that may deplete energy reserves). These cohorts (i.e., groups of ticks of similar physiological age) will react differently to weather conditions because their physiological age is different, producing disparate statistical results when correlating “weather” with “tick abundance”. Therefore, researchers usually collect ticks at monthly intervals, aiming to explain their density with factors derived from weather, hosts and/or landscape data, obtaining variable success modeling the tick density at different time periods [5]. However, the proportion of both “old” and “young” ticks will change along with the time slices of the surveys, allowing different combinations of explanatory variables. Older ticks, with higher physiological stress, will probably show a stronger correlation with long-term weather values while newly recruited young ticks, with low physiological stress, will probably show a more prominent response to short-term variables [6]. It should be noted that a theory must explain everything with the same combination of explanatory variables—a theory that switches variables according to time slices does not conform to this requirement. Unfortunately, it is not possible to ascertain which ticks belong to different cohorts in a direct way [7], but there are methods to evaluate the physiological age of a tick (see below). As far as has been demonstrated, the simple accumulation of temperature and the deficit of water saturation (and their combination) may be the best explanatory variables [7]. However, extreme weather events or the aggressiveness of the target tick could interfere with the correct evaluation of these statistical calculations, which differ among species and for which a general rule cannot be provided. The importance of “permanent collectors” carrying out periodical surveys emanates from these concepts.

### 2.2. The Challenges of Field Collections

Our view is that to gain wide-area comparable data, there are, at least, four main issues that need to be addressed in field surveys of exophilic ticks.

#### 2.2.1. The Repeatability of the Measurements of Tick Density

Multiple track surveys at the same site at the same time should provide similar data on tick density. We do not know what proportion of ticks are “extracted” from the vegetation nor whether the survey methods (dragging, CO_2_ traps, etc.) manage to obtain a representative number of ticks. Differences in vegetation height or in the shrub layer can cause differences in extractability. The evaluation of the reliability and reproducibility of dragging is something rarely addressed [8]. Furthermore, the length of the track should be flexible and related to the number of ticks collected, i.e., low densities of ticks in a site might require longer tracks to increase accuracy [8]. In summary, we strongly recommend carrying out a pre-survey in which collection effort is evaluated (according to published methods [8]) to determine the limitations and to estimate the best reproducibility of results.

#### 2.2.2. The Evaluation of the Physiological Stage of a Tick

The physiological stage (or “tick physiological stress”) is an evaluation of the fat reserves of a tick; the lower the reserves are, the “older” the ticks are in physiological terms. It is not a measure of age in calendar days but an estimation of how the tick spent its energy resources due to adverse events (i.e., low humidity or any environmental factor that depletes tick reserves). It is the manner of determining if a tick is “young” (probably newly molted) or if it has already spent a long time questing in the vegetation, thus depleting energy resources [9]. The classic method is to measure the fat contents of the tick as a proxy of its energy reserves [2,9,10]. This is an effective but time-consuming procedure. The simplest approach is based on the dry weight, preferably in combination with a scutal/dorsal index; this approach has been adequately addressed [11] and morphometric age ratio can also be obtained from living tick specimens.

We propose the development of methods to calculate the tick’s physiological age based on molecular biology. Previous studies addressed the degradation of nucleic acids from host blood [12,13], a field that has been since neglected. Once it has been established that the Cytochrome *b* (*cytb*) gene from hosts degrades at a given rate [14], qPCR methods could detect the number of copies of this gene present in the remnants of a host’s blood in the tick as a measure of a tick’s physiological age (older ticks are those with lower levels of *cytb*) and then establish the proportions of old and young ticks in every survey. However, for *cytb,* the starting load may depend on the host’s health status and the species of vertebrate involved. These methods could be compared to a simpler approach based on the dry weight, preferably in combination with a scutal/dorsal index to achieve a comparison of methods. We hope that both morphological and molecular methods will come together in the near future, providing a quick and accurate determination of tick physiological age.

#### 2.2.3. Proposals for Weather Recording

Some studies use weather data from the “nearest climate station”. However, that climate recording station may be dozens of kilometers away and, moreover, climate data are often recorded well above the vegetation canopy. It is preferable that temperature and humidity data loggers be used for recording time series of weather, starting preferably well before tick dragging [15]. Data from different surveys at different temporal resolutions and without weather recorded by data loggers are far from comparable in future meta-analyses. Data should include hourly time intervals to record minimum and maximum temperature and humidity (or a measure of water contents in the air), allowing the calculation of the accumulated temperature resulting from the values before and the saturation deficit of water (or the relative humidity). We think that daily data (coming from average or the accumulated values of hourly intervals) should always be used, even if monthly surveys are carried out. These datasets, at daily intervals, should always be included as supplementary material and attached to the manuscript to provide context.

Long-term averaged climate data (i.e., averages for a period of several years) should not be used to explain current tick abundance since historical conditions of climate do not necessarily reflect current weather. Many unresolved questions on the impact of climate on tick abundance and seasonal activity could be addressed with this type of information aimed at meta-analyses. It should be noted that if weather features are not recorded well in advance of the first survey in a study, researchers will miss about six months of explanatory variables for questing activity.

#### 2.2.4. The Systematic Description of the Vegetation in Which Surveys Were Carried Out

A coherent description of the vegetation layer in which surveys were carried out should be provided with results of tick surveys. It is not enough to state that the vegetation was “short” or “tall” because a standard denomination of the botanical associations would probably result in a better understanding of the “landscape” in which collections were made. This is however an issue since the phytosociological “schools” in the world use different classifications. On the other hand, the widely available datasets describing the vegetation for large areas like continents cannot capture the small-scale tick habitat associations. For example, the category “Caducifolious forest” alone is not suitable for describing every kind of plant associations in which different species of ticks can be found, associated with particular groups of vegetal alliances commonly found in a Caducifolious forest.

There is a harmonized description of phytosociological associations for Europe [16] presenting a comprehensive, hierarchical system of alliances, orders and classes. See Appendix A for examples. A specific community of plants is considered a social unit, the product of conditions that allow the presence/absence of plants that naturally occur together and that results in its phenology. Therefore, the use of such comprehensive alliances would greatly improve the description of the vegetal layer and its phenology because there is a biological and ecological meaning behind each association. This approach has been successfully used [17,18,19] to describe the associations of several tick species with coherently described botanical associations.

The hierarchical classification for Europe mentioned above [16] is homogeneous and gives a coherent description of the vegetal cover. We are not aware of similar classifications for the rest of the world. In the USA, this concept was largely rejected promoting the study of the environment impacting the species, while specific associations of plants occur randomly because of individual preferences and responses to gradients. Even various European countries have different standard denominations (as summarized in [16]). We believe this issue does not yet have an easy solution of applicability for the collection of ticks and would recommend a consensus opinion by tick researchers about this neglected topic.

#### 2.2.5. Other Issues Associated with Collections of Off-Hosts Ticks

There is a lack of standardization of methods for quantitatively collecting questing ticks of some species that are not easily captured by flagging, such as those belonging to the genera *Amblyomma* or *Hyalomma*. Commonly, these ticks are picked up from the ground by hand by researchers (a very low efficiency method with has irregular results because it depends on the training of researchers), or, in some cases, using CO_2_-operated traps or pheromone operated decoys. Since researchers may have different levels of training for collecting ticks, results from multidisciplinary, multi-team or multi-site projects may be difficult to compare.

We propose prioritizing efforts to develop reliable and repeatable methods for collecting ticks that are not well captured by standard flagging/dragging. These efforts could be primarily directed at performing release-recapture studies. For example, ticks could be marked with a spot of paint on the dorsum, released and then recaptured using different methods in order to determine their efficiency. Similar problems exist for endophilic ticks, which live inside the burrows of vertebrates. The efficiency of CO_2_-operated traps placed at the entry of these shelters has not yet been extensively studied [20]. 

## 3. The Reporting of Prevalence of Ticks on Hosts

**Figure 2:**
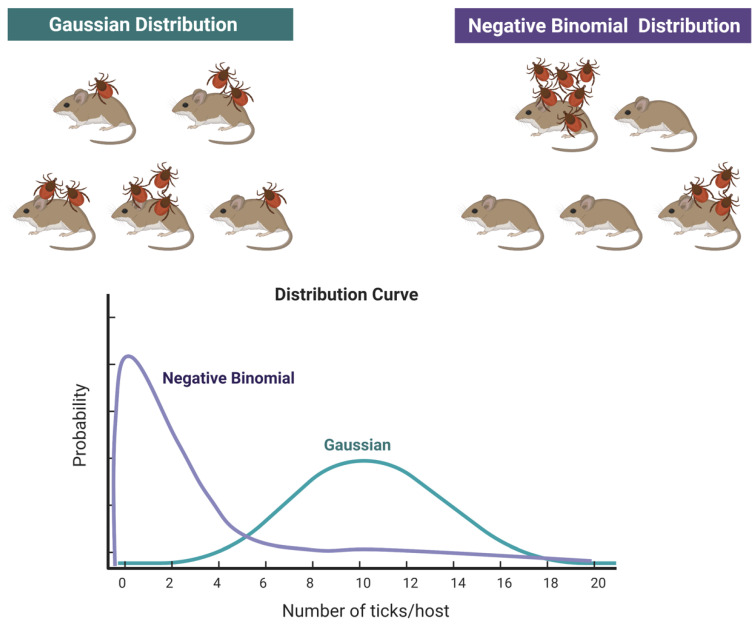
The scheme shows a Gaussian-type distribution of a species of tick on a population of hosts, with every host carrying ticks and a variable number of ticks on each host, and a negative binomial distribution, showing how most ticks are concentrated on a few hosts, while most hosts do not carry ticks at all. Both distributions are displayed as a chart. Mean number of ticks and standard deviation are the same for both distributions. Created with BioRender.com.

Much of our knowledge about ticks is from specimens collected while they are feeding on hosts. This is not a method appropriate to measure tick abundance because preferences for a given host and individual susceptibility of each vertebrate to carry ticks bias the data obtained [2]. Data of “abundance” of ticks on hosts (even if considered as “sentinels”) cannot be used as reliable markers of the actual abundance of ticks, nor as a measure of “risk”. However, sentinel animals can be excellent indicators in presence/absence studies. Data on the abundance of ticks on vertebrates is usually reported as the mean number of ticks found on vertebrates, together with the standard deviation of the mean. This intends to provide a view of the relative importance of different vertebrates as hosts for ticks, the seasonal variations of the parasitic load or is linked to other epidemiological issues like the presence/absence of pathogens. However, these values are disturbingly equivocal, because the distribution of ticks feeding on hosts does not follow a Gaussian distribution [21,22] but a negative binomial distribution (or aggregated distribution [23]). In a Gaussian-like distribution, ticks are distributed over the population of vertebrates around the mean value, each host carrying some ticks, with low variance. However, fieldwork on tick-host relationships has demonstrated that the frequently observed clumped distribution of ticks on hosts can be described better by a negative binomial distribution, following pioneering work [24,25]. This leads to the paradoxical situation that the reporting of the mean number of ticks on a population of hosts provides no reliable information, even if every tick is collected (see Figure 2). Charts in Figure 2 show how the total number of ticks collected on a given number of hosts is the same but in the case of a negative binomial distribution, most of the hosts carry no or very few ticks, and a few hosts carry most of the ticks.

A comparison [26] of these indices of aggregation found that the estimate of *k* (an inverse measure of aggregation) from the negative binomial distribution varied least with mean parasite load and sample size, and this is now the index of aggregation most used by epidemiologists. Not only are the vast majority of parasite datasets best described by the negative binomial distribution [25,27,28], but its exponent *k* is used to capture parasite over-dispersion. Methods to calculate the *k* values of distributions of parasites on hosts have already been published [29]. Measures of aggregation, together with a broad explanation of the factors leading to aggregation of macroparasites on hosts have been explained and compared [29]. However, the *k* exponent has been criticized [23], and a new measure of aggregation called the Index of Discrepancy (*D*) has been proposed. The Index of Discrepancy (*D*) [23] is represented as the discrepancy between the observed parasite distribution (denominator) and the hypothetical distribution in which all hosts are equally parasitized (numerator):

$$D=1-\frac{\sum_{i=1}^{N}\left(\sum_{j=1}^{i}x_{j}\right)}{\sum_{i=1\;}^{N}\bar{x}i}$$

where *x_j_* is the number of parasites on host *j* (hosts are ranked from least to most heavily parasitized) and *N* is the total number of hosts in the sample. Therefore, the denominator estimates the average value of the parasites in the sample of hosts. *D* may range from “0” (representing no aggregation) to “1” (representing maximum aggregation with all parasites on one host). We consider that the simple reporting of mean and dispersion values (i.e., standard error) of ticks on hosts is a simplistic approach that does not contribute to disentangling the factors affecting the distribution of ticks on hosts, like age, sex, weight, etc., as already addressed [30].

Several factors may affect the ability to determine the actual prevalence and abundance of a tick species on a vertebrate population. The most obvious are the sex and age of the hosts. In this regard, it must be emphasized that, when studying field-trapped wildlife, some age or sex groups are often over-represented. Furthermore, physiological condition can affect both the capture probability and the tick burden of a vertebrate. Other factors include the areas of the body surveyed [31], the experience of the researcher or the time expended looking for ticks, something that should be adequately evaluated for the host-tick pairs existing in an area. For example, between 5 and 20 min have been recommended to estimate the total number of adult fleas on an animal [32]. Similar estimations should be standardized for ticks and the associated vertebrates. 

Studies reporting the statistical distribution of ticks on the surveyed hosts should include the simplest values like mean and a measure of dispersion in the body of the paper. However, we strongly recommend the publication of the actual number of ticks on each individual host to allow other researchers to evaluate the aggregation of the ticks on hosts. This immediately allows large area comparisons across similar studies. An explicit indication of the species of ticks and vertebrates, the stages and number of ticks found on every single specimen should be included. Data on hosts that do not carry ticks is a must. Promoting the publication of host-by-host raw data is the best way to harmonize studies.

## 4. The Reporting of Prevalence of Pathogens in Ticks

Data on infection rates by pathogens in ticks came from two main sources, namely surveys of off-host ticks and assessment of the pathogen’s presence in feeding ticks. The former is obtained through field surveys of questing ticks, with ticks processed individually or in pools and obtaining a “snapshot” of the prevalence of ticks at the time the survey was carried out. In some cases, studies are continued through time to achieve a “movie” instead, since the potential variability of prevalence values in the same region over time is expected. We want to demonstrate (point 4.2) that a value of “prevalence” in feeding ticks is an unreliable index about which both researchers and reviewers should be aware when publishing.

### 4.1. Reporting Pathogen Prevalence in Questing Ticks

Processing questing ticks to calculate the prevalence of pathogens can be addressed by either using individual ticks or pools of ticks for DNA/RNA extraction. Pools are commonly used to reduce cost and workload. Other than the usual procedures for safe extraction of nucleic acids, a few rules should be strictly followed:

- Pools must contain only the same species and stage of ticks.
- A method like the Minimum Infection Rate (MIR) [33] can be used to assess the prevalence if working with pools.
- Individual details of each pool or individual ticks should be included in a supplementary file attached to the publication, to provide context.

**Figure 3:**
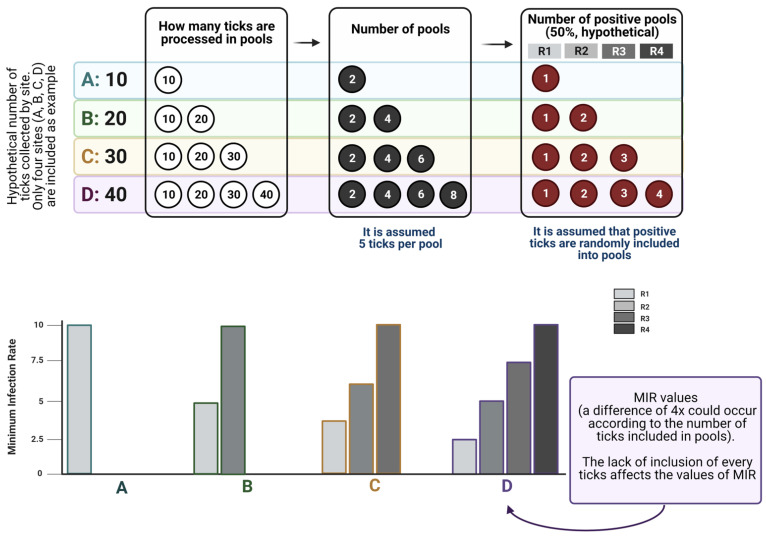
The effect of tick sampling on hosts on the determination of the Minimum Infection Rate (MIR) of a hypothetical pathogen. In table A we introduce four hypothetical host specimens (A, B, C, D) that carry 10, 20, 30 or 40 ticks. Of these hosts, researchers can retrieve all or a fraction of these ticks (indicated in the columns) and pools are prepared assuming a fixed number of 5 ticks per pool. We also assumed a fixed positivity rate of 50% under every condition, and made assumptions about the ways the calculation of MIR is affected by these factors. The chart shows the values of MIR obtained from hosts A, B, C or D based on the number of positive pools. It can be observed that values can change from MIR = 2.5 to MIR = 10 according to the number of ticks collected, assuming the same infection rate. The conclusion is two-fold: even if researchers consider MIR from ticks collected while feeding to be reliable (which it is not), it is deeply affected by the number of ticks collected. Created with BioRender.com.

The first two points have been commonly adhered to in recent reports on the topic. However, the preparation of pools may have a serious impact on the obtained results, and we highly recommend the reading of studies on the use of pools of different sizes to understand how these indexes work [34]. It must be noted that MIR is commonly used to test large numbers of randomly collected samples, but not a subset of samples. The MIR assumes that there is only one infected tick in each positive pool. Rather than estimating actual infection rates, MIR measures the lower bound [34]. The use of MIR may greatly underestimate infections by pathogens in ticks in situations when prevalence is high and/or pool size is large [35]. Therefore, we propose that MIR be used when a low infection rate is expected in ticks (around less than 2%); otherwise, it may be worthwhile to test ticks individually. We recommend that all collected ticks be processed, instead of preparing pools with “some randomly selected ticks”. Figure 3 displays how MIR is affected by the lack of inclusion of every tick in pools, exploring how MIR changes across scenarios with variable numbers of ticks processed, and how probability laws operate on the allocation of infected ticks into the pools. The data shown in Figure 3 demonstrate that the inclusion of a variable number of ticks in the analyzed pools leads to contradictory results in the calculation of MIR, therefore leading to a biased estimation of the prevalence of a pathogen in questing ticks. 

We are not aware of studies investigating the distribution of pathogens in the individuals of a population of questing ticks; in other words, supplementary material allowing comparative analyses is rarely provided with a manuscript, with obvious exceptions. Two important points should be addressed, namely: (i) How many ticks are sufficient to detect a pathogen with reasonable statistical power? And (ii) how should the effects of over- or under-sampling in the calculation of MIR be evaluated? A method to address these questions has been proposed [36]. The method is focused on mosquitoes and viruses but can be directly translated to ticks and tick-borne pathogens. A method for calculating minimal sample size, with applicability in tick research, was proposed in the same study [36].

Under-sampling is believed to be a significant obstacle in developing robust prediction results for MIR of pathogens in ticks. Under-sampling could be caused by not having enough transects within the geographic area of study, not testing all ticks collected, low tick abundance or a combination of these factors. Some of these are outside of the control of researchers, like low tick abundance, but an exploratory tick collection can be done to estimate how much effort is needed to obtain an adequate sample size. The methods mentioned above [36] could be applied to the calculation of the effect of under-sampling in collections of questing ticks and detection of pathogens. Another, perhaps more practical method addressing the distortion of MIR values by under-sampling was proposed [37] using the so-called “Value of Information” (based on [38]). The aim of this study was to use available data on West Nile virus detection in mosquitoes in two counties in the USA to detect the effect of random data removal on the calculation of MIR. This was aimed to detect a threshold producing an impact on estimations, after the artificial and random removal of samples estimating a “minimum sample size”. The study also provided an algorithm by which MIR error can be estimated. According to these authors, this could provide researchers with an estimate of the sample size needed to avoid unreliable results.

A similar procedure could be envisaged for tick surveys: what information is available about a territory and how could the random removal of information affect the calculation of MIR? We would like to stress that previous recommendations immediately address the need for availability of raw data to researchers, something currently unavailable for many reports on the topic. As repeatedly mentioned, we aim for wide area meta-analyses, which are impossible to conduct without raw data.

### 4.2. Reporting Tick-Borne Pathogens in Feeding Ticks

There is a trend to report values of infection rates of pathogens in feeding ticks, either because it is more efficient to obtain ticks from hosts than through flagging or to implicate vertebrates in pathogen life cycles. We would like to stress that, when used on its own to gain information about the “risk of pathogens”, this is an unreliable procedure that only adds noise to studies on ticks and that should be systematically avoided. We aim to demonstrate that the calculation of a pathogen’s prevalence on collections of feeding ticks is unrealistic, unrelated to the actual rates in questing ticks and provide a biased view of the actual infection rates in the field. The most common mistake is the recurring fallacy that the DNA/RNA of a pathogen found in a feeding tick may be equated with the tick’s ability to transmit the viable pathogen, ignoring the fact that the genomic material might be acquired from the blood of the vertebrate. We have already written about this topic [39] but, unfortunately, the contrary opinion seems to have gained position in the publication pipeline. Collections of feeding ticks, in which the DNA/RNA of a given pathogen was detected, are sometimes reported to be a “new vector” of the pathogen (see below in Section 6). Moreover, adult ticks retrieved from some hosts may have acquired the pathogen in an immature stage parasitizing a different host. For example, the detection of DNA of *Rickettsia massiliae* was reported in several tick pools retrieved from carnivores, but all the hosts were negative for that pathogen [40]. Therefore, those ticks most likely acquired the pathogen from hosts of the immature stages (e.g., rodents), and not from the carnivores.

Ticks feeding on infected vertebrates could acquire a pathogen at variable rates depending on many variables [39]. Ticks of different stages of engorgement could have a different probability of acquiring a pathogen (a longer feeding time could lead to a higher probability of the tick being infected). However, this is also influenced by the different abilities of the reservoirs to transmit a pathogen and of ticks to acquire it. Fed ticks are commonly processed in pools because of the obvious logistic and economic difficulties in processing them individually. Therefore, the issue is that a pool may consist of ticks that were already infected before feeding together with ticks infected at variable rates while feeding. This violates every statistical and biological assumption about the rates of infection of pathogens in ticks. 

**Figure 4:**
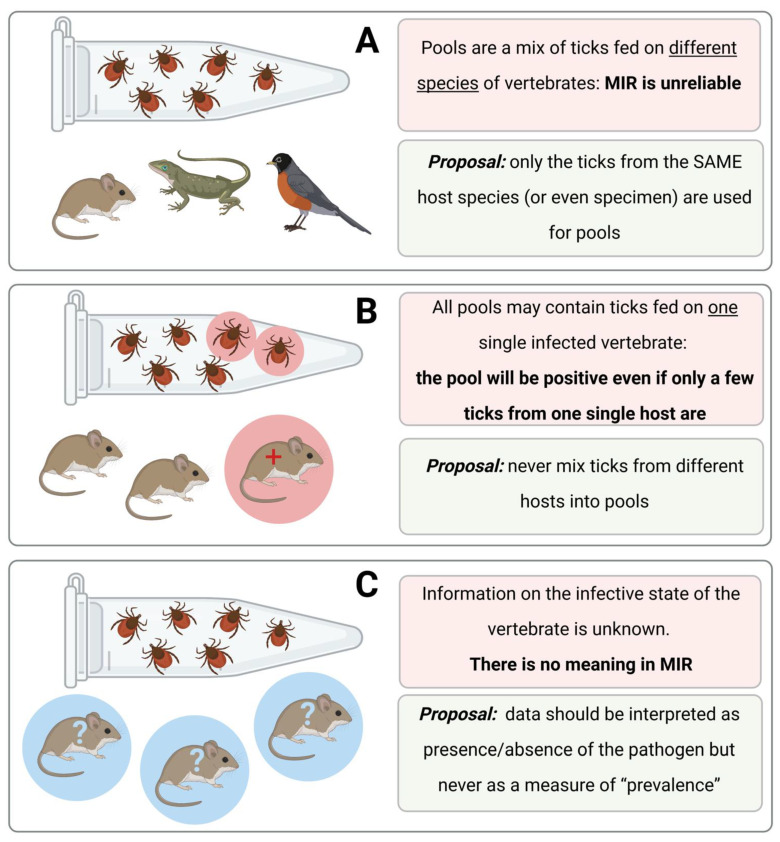
The inaccuracies commonly reported when estimating the Minimum Infection Rate (MIR) of a pathogen detected in ticks collected while feeding. In (**A**), ticks are collected from different species of hosts and pooled together. We believe that unreliable conclusions would be drawn from these data, since there is no way to determine which host (if any) contributed to the presence of pathogen-derived DNA in the feeding ticks. In (**B**), ticks are collected from the same species of host, but only one host is contributing to the presence of the pathogen; pools contain material from different individuals. If pools are random, ticks coming from the only positive host could “contaminate” every sample giving an overestimate of the infection rate in ticks. In (**C**), the most commonly reported situation, the infection status of vertebrates is not recorded. We recommend not to use MIR values based on collections of feeding ticks. These data may demonstrate the presence of a pathogen in a site but should never be used in a quantitative way. Created with BioRender.com.

Moreover, it is not possible to correlate the values of prevalence in questing ticks with infection rates in feeding ticks. Vertebrate hosts may differ in their ability to transmit a pathogen to feeding and ticks may differ in their ability to take up the pathogen. Several published studies even evaluated “statistical differences” among sites or pairs of vertebrate-tick species and “demonstrated” that the rate of infected ticks in a region was significantly different than in other regions, using only data from feeding ticks. It must be realized that the values obtained tend to artificially increase the actual prevalence of a pathogen in ticks, since several pools may come from ticks collected on the same infected reservoir, producing inflated values of infection rates. Figure 4 shows hypothetical situations, which are commonly mentioned in published papers, but that do not give reliable information about the infection rates of a pathogen in ticks.

It should be noted, however, that data about pathogens obtained from feeding ticks may be the only opportunity to gain information about these pathogens. For example, this may be the case for rare species of ticks or in sites where ticks are thinly spread and flagging is impracticable. Under these and many other unusual circumstances, data about infection rates of pathogens in ticks could provide a necessary minimum amount of information if researchers adhere to a basic protocol, as follows: (i) never mix ticks in pools from different host specimens, (ii) always inspect the infection status (+/−) of the host, (iii) publish these extra results demonstrating the positivity status of each individual host and the ticks collected from it and (iv) observe the rule that these data explain the local situation and are not comparable to other data in either time or space.

A completely different question is raised in trying to understand the fine relationships between vertebrates, ticks and pathogen circulation. As an example, a comprehensive review of the uncertainties regarding the dynamics of *Borrelia burgdorferi* s.l. was published [41]. These authors explicitly stated the need to predict the nymphal infection prevalence from vector-host-pathogen interactions, which “requires data on the fraction of larval ticks that feed on each host species, the fraction of hosts of each species that are infected, and the reservoir competence of these hosts for transmitting *Borrelia* spirochetes.” [41]. In aiming to do this, a protocol should be adhered to, including the processing of ticks individually along with details of the infection status of the host on which ticks were feeding. Data should be reported with details for each vertebrate separately, stating how many ticks have been found on each vertebrate, the status of each feeding tick (+/−) and the status of each vertebrate. Following the aim of this review, we propose including a coherent supplementary file that addresses all of these issues. We firmly believe that this would be an effective way to gain information about these complex relationships, that could be expanded with future, local/regional projects. The exploration of the data from many sites would provide a more complete overview of these fine details, without the need to “begin from the beginning” every time.

## 5. GenBank, the Molecular Identification of Ticks and the Bona Fide Sequences

Across the entire phylogeny, a similar set of core gene regions, such as the nuclear internal transcribed spacers (ITS), ribosomal RNAs (e.g., 12S, 16S and 18S) and protein-coding genes from the mitochondrial genome (e.g., *cytb*, cytochrome oxidase subunit I and II [*COI* and *COII*] genes), have proven particularly useful for taxonomic discrimination. GenBank was borne as a data repository of molecular sequences of every living organism. The Barcode of Life DataSystems (BOLD) (https://v3.boldsystems.org, last accessed on 1 February 2021) and GenBank (https://www.ncbi.nlm.nih.gov/nucleotide/, last accessed on 2 March 2021) are two of the main public databases of DNA data for animals, plants and fungi. BOLD currently (March 2021) contains sequences for ~296,000 formally described species (~7 million specimens). For a sequence to obtain a formal barcode status in BOLD, several elements must be provided: species name, voucher data (storing institution and catalog information), collection record, identifier of the specimen, sequence of >500 bp, primer information and the raw sequence data files. Note the emphasis on “voucher specimens”. Once uploaded, BOLD administrators perform quality checks of data prior to making it public (i.e., confirmation that the sequence is not that of a contaminant, is a true functional copy and is of adequate quality). GenBank is much larger and contains >212 million sequences. GenBank also performs basic quality checks on all new submissions, such as vector contamination, proper translation of coding regions, correct bibliographic citations and correct taxonomy. However, unlike BOLD, GenBank is just a sequence repository, not a curated sequence library. 

Nucleic acid sequences stored in GenBank are mainly used by tick researchers for two purposes: (i) the confirmation of the morphological identification of ticks (or to proceed to a direct molecular identification) and (ii) the production of phylogenetic trees of organisms allowing a comparison of newly reported specimens/strains. These procedures have become mandatory in the descriptions of new species of ticks or to confirm morphological identifications. There is no complete consensus about which genes best represent the variability of the target organism, and it seems that some genes could work better for some species, i.e., the same set of genes does not necessarily have the same reliability with different species of ticks or pathogens. Recent advances toward the complete mitochondrial genome of ticks, supported by increasingly cheap and fast sequencing technologies, are providing an unexpected view of the phylogenetic relationships among tick taxa and are challenging previous views of the phylogeny of ticks. Important contemporary reports on the topic can be found in [42,43,44,45] to cite a few.

Even if the complete mitochondrial genomes are available for several species of ticks, researchers must cope with the issues of molecular identification of ticks, using the “classic” approach based on the available sequences of a few genes. These approaches to identification or to place newly collected ticks in a phylogenetic tree should take account of the reliability of the sequence, which depends on the reliability of the identification of the specimens from which the sequences were obtained. However, published sequences from GenBank are in most cases downloaded randomly in the hopes that “a number” of sequences will be sufficient to fill a tree. Consider the situation in which a tick has been unreliably identified, its molecular sequence uploaded and/or published, without the availability of voucher specimens. The use of sequences from unreliably identified ticks will produce “noise” in any further analysis: the target specimens included in a phylogenetic tree made up with unreliable sequences (i.e., wrongly identified ticks) will bias conclusions. For example, a recent study demonstrated that the accuracy of identification of insects based on GenBank sequences was 53% [46].

A previous study [47] demonstrated that European experts in tick taxonomy (14 independent laboratories) failed more than 40% of the times to accurately identify ticks, producing misidentifications at the species level, when working with unlabeled samples submitted to each laboratory. Interestingly, molecular methods of identification performed better than morphological ones. As it currently stands, the detection of anomalous and suspicious records of either ticks or pathogens in GenBank is needed as it is still the best source for tick identification. At issue is the choice of the right sequences. As a starting point, we propose some golden rules that should be adhered to when submitting or using sequences from GenBank, as follows:

*a*. Representative tick specimens even when not describing new species, should be made available, if possible. These specimens should be deposited in a widely acknowledged international tick collection, facilitating the exchange of material. The voucher specimens could then be examined in case of concerns with the sequence(s) obtained from them. The DNA extraction should be done ensuring maintenance of the specimen in the best possible condition, avoiding the loss of morphological structures of interest. The DNA can be extracted (i) from a single leg leaving the rest of the specimen intact or (ii) making a cut in the lateral third of the idiosome and collecting the exoskeleton after incubation and lysis of tissues.
*b*. The search for downloadable sequences in GenBank is not a random selection. Researchers should use sequences coming from (i) type or neotype specimens of (re)descriptions of species and with voucher specimens available, (ii) specimens for which a complete gene has been sequenced, to obtain more phylogenetic information, (iii) specimens for which reliable illustrations exist in the original publication allowing an unbiased identification of the tick in case of concerns. It would be necessary to mark these *bona fide* sequences as gold standards for future research.
*c*. Always use the complete gene sequences. Do not use sequences from “species yet to be characterized” or that are striking records far from the known range of the target species. This is not a rejection of these records, but a cautionary comment about their indiscriminate use before the data is firmly established as reliable.
*d*. If available, include the geographical coordinates of the specimen when submitting sequences to GenBank. The name of a country alone associated with a sequence is insufficient for further meta-analysis. Phylogeographical studies could be developed if coordinates are included, associating sequences with other factors that could drive the disparity observed in the tree. This is something yet unexplored in many fields of ticks and transmitted pathogens research, despite the increasing number of sequences with coordinates. The preferred method, other than submission to international repositories, would be to publish the sequence as supplementary material to placing it in context with the contents, findings, and available data.

In any case, cleaning up the sequences available in GenBank for ticks is urgently needed. We believe that preliminary steps could be carried out by computer algorithms (align, cut and assign an index of reliability to literally hundreds of thousands of sequences). Then, the assignment of the actual status to a sequence could be done manually. Our proposal is a network of national reference laboratories for tick and pathogen identification. These laboratories should focus not only on the identification of ticks and pathogens submitted by researchers under confidential protocols, but also on providing advice for future surveys and the “how-to”. Efforts by that network could result in a dedicated website, including reliable sequences for both ticks and pathogens, manually curated by experts and including voucher specimens, aiding researchers willing to carry out comparative studies. Given the increasing importance of ticks, this should be a priority in the policies of human and animal health.

## 6. Evidence for Vectorial Competence of Ticks

Even though the issue has been previously discussed [39], one of the mistakes that still appears in many publications is assigning “new vector status” to a tick when the DNA/RNA of a pathogen is detected, even when they are found feeding on hosts, as mentioned above.

As discussed, ref. [39] confirming that a tick is a vector of a specific pathogen requires evaluation and confirmation that (i) the tick has the capacity to become infected by feeding on infected hosts, (ii) the fed tick can maintain the pathogen after molting to the next life cycle stage or the F1 generation and (iii) the molted stages of the ticks can transmit the pathogen to an uninfected host while feeding. These criteria are exceedingly difficult to demonstrate using only field surveys and require experimental data. However, there are different degrees of evidence that could allow speculations about whether a tick could be a competent vector of a pathogen. These are based only on pure field findings. The “degrees of evidence” that could be obtained from ticks while feeding are as follows:

- Null evidence: detection of pathogen DNA/RNA in a fed tick (or a pool) retrieved from hosts; these data only create noise in the corpus of research. Records could be used for reporting presence/absence of a pathogen in a site (i.e., using the ticks as “sentinels”). Its support for further conclusions is null.
- Spatial overlap of vector, vertebrate and pathogen distributions: there is a statistical association of the spatial distribution of the three actors. For example, the distribution of *Cytauxzoon felis* in the USA overlaps the known distribution of the tick *Amblyomma americanum* and the host, the bobcat (*Lynx rufus*) [48]. The association is statistical, demonstrating that prevalence was significantly higher in sites with established populations of *A. americanum*. Collectively, these data suggest that the bobcat is a natural host for *C. felis* and that *A. americanum* is likely a prominent vector.
- Presence of a single tick species associated with a high prevalence of a pathogen in a host population (or in high loads relative to other species): this may be indicative of a tick circulating the pathogen to the hosts, but also that the tick acquired the pathogen while feeding on other hosts in previous stages of its life cycle. Results should be interpreted with caution. For example, a high prevalence of a *Hepatozoon felis*-like strain in a grey fox (*Lycalopex griseus*) population in Argentinian Patagonia was reported [49], and *Amblyomma tigrinum* was the only tick species found on those foxes. Thus, a potential role of *A. tigrinum* as a vector of *Hepatozoon* is suggested, with further evidence needed to confirm this.
- Detection of pathogen DNA/RNA in ticks retrieved from hosts, after bloodmeal digestion and molting: this is proof that at least remnants of the pathogen’s DNA/RNA persist in the tick after molting. It is not a clear demonstration that the complete and infective pathogen is still in the tick after molting but warrants future studies on the system.
- Detection of pathogen in tick salivary glands: the pathogen managed to survive the molting instar and migrate to salivary glands of the tick. This is an indication that the system deserves special attention.
- Observation of mature parasite stages in the tick (i.e., the sporogonic development of some *Hepatozoon* sp.). For example, evidence has been provided of sporogonic development of *Hepatozoon canis* in specimens of (reported as) *Rhipicephalus turanicus* ticks collected from a naturally infected fox from southern Italy [50]. In the case of some *Hepatozoon* sp., the tick must be ingested by the host to complete the cycle. Therefore, in ticks (definitive host) the cycle is completed when gamont develops, reproduces sexually, and matures into sporozoites.
- Full evidence: Experimental assay involving infected vertebrates feeding naïve ticks, detection of the pathogen in fed and molted ticks, allowing these newly infected ticks to feed in naïve vertebrates and detection of the pathogen in the latter. This has also been referred to as “xenodiagnosis”. This is final proof of the circulation of a pathogen by a tick. However, a contrasting view is that “what happens in the laboratory, does not necessarily happen in nature”. The establishment of vectorial ability must be ideally based on both laboratory and epidemiological evidence [51].

## 7. Conclusions

This opinion paper aims to encourage data harmonization regarding reports on ticks and tick-borne pathogens and the publication of raw material as obtained in surveys and proposes the observation of a minimal set of rules supporting the reliability of data. We consider the publication of raw material a necessary step for wide-area meta-analyses. Researchers studying ticks and tick-borne pathogens should realize that we will never manage a wide-scale experiment capturing the complexities of relationships among climate, landscape, vertebrates, ticks and pathogens, and must depend on comparable data to derive reliable conclusions. This review is a plea for homogenization and publication of all available supporting material obtained in field surveys on individual ticks and/or vertebrates. This document is not a consensus opinion reached by many specialists in the field and is open to debate; it only reflects our view about how data should be communicated in mainstream journals covering the topic.

We aim to describe the complexities behind serendipitous correlations between the density of ticks and climate variables, providing a minimum set of rules that should be observed. A minimum protocol for obtaining weather data and collecting ticks and the inclusion of every raw variable in the published report are necessary for these wide-area analyses. We call for the inclusion of raw data in these reports because they can be useful in future comparative studies. No matter the statistical comparisons, the raw data will always help these further analyses.

We stress the need to develop methods for reliable and repeatable collections of ticks that are not easily captured by standard flagging. The community of tick researchers tends to focus on a few species and forget others that are crucially important in animal and public health. It is striking that, while we can capture the dynamics of i.e., *Ixodes ricinus*, we cannot calculate the density of questing *Hyalomma* spp. or some *Amblyomma* spp.

We recommend adherence to a standard *corpus* when explaining vegetation alliances, even while we recognize that this topic still requires harmonization by botanists. This has always been recognized as an important feature delineating tick-host relationships in field surveys. A long and subjective narrative is commonly used to describe the vegetation in which surveys have been carried out, rather than using internationally recognized standard terms.

This manuscript is an appeal for reporting the prevalence of tick-borne pathogens in proper terms, avoiding conclusions based on simple molecular findings that do not reflect ecological associations. We would like to promote the publication of the raw data obtained in field surveys regarding vertebrates, ticks and pathogens, in the form of supplementary data associated with the article, allowing future meta-analyses. Without these datasets, researchers cannot compare prevalence values. We recommend the inclusion of these datasets in the context of the publication, and/or uploading them to online repositories (however, the context is missing in the latter approach). Obviously, the rules for each journal will dictate the procedure to follow. We strongly believe that separate data for results of each vertebrate and tick is a must if we aim to achieve the complete picture. The mean and the standard error, as basic values published in the body of the paper, are not enough to understand how ticks behave.

Finally, we discuss the uncritical use of “randomly chosen sequences” from GenBank to support identification of ticks or pathogens. The selection of sequences from GenBank must be an elaborated protocol, using only the sequences that are gold standards, because these sequences were obtained from voucher specimens or there is literature confirming their reliability. Some sequences have already been used in other studies and are validated as bona fide data. We firmly support the development of a dedicated server of curated sequences of ticks and transmitted pathogens that could aid future research.

We recommend a properly designed study based on the research question and not the other way around. If the study aims to demonstrate the vectorial capacity of a tick, a molecular survey of feeding ticks will never answer the research question. We also discourage vacuous conclusions that are often found in articles on tick-borne pathogens stating that some tick species could be considered a vector, or some host as a reservoir, just based on the finding of genomic material of a pathogen in ticks.

## Data Availability

Not applicable.

## Appendix A. Definitions

- Aggregated (Negative binomial) distribution: a distribution of parasites or pathogens in which few individuals host high burdens, whereas most individuals host few or no parasites.
- Cohort: a group of ticks that moult and quest in the same period of time and therefore have a similar physiological age.
- Data logger: a device that measures and records data (e.g., temperature and humidity) at defined intervals.
- Gaussian distribution: a distribution following a bell-shaped curve with an equal number of measurements above and below the mean value. It is also known as Normal distribution.
- Minimum Infection Rate: the ratio of the number of positive pools to the total number of ticks (or other vectors) tested for a given pathogen (abbreviated as MIR).
- Poisson distribution: a probability distribution that expresses the probability of a given number of events occurring in a fixed interval of time or space if these events occur with a known constant mean rate and independently of the time since the last event.
- Physiological age: the environmental stress that a tick has supported since its last molt (or hatching); it is directly related to their energy reserves and how they are depleted. It commonly depends on accumulated temperature and accumulated saturation deficit, two variables that can be easily measured with data loggers.
- Phytosociology: a botanical discipline that describes the diversity of plant communities and the environment of a given territory, in terms of associations of plants. It is a hierarchical definition of vegetal species that tend to appear together because of climate gradients and soil conditions. It is extensively used in Europe and provides an unbiased classification of the cohort of vegetal species occurring together. For example, the Tilio-Acerion is “an alliance of sub-montane maple and lime woods of humid ravines from among the mixed broadleaf woodlands of more fertile soils. It typically has a diverse canopy of trees and a rich ground flora of herbs, ferns and bryophytes dependent on nutrient-rich moist soils”. The meaning of the i.e., the alliance “Pegano-Harmalae-Salsoletea Vermiculatae” may be hard to capture by tick researchers, but it is defined as “Thermomediterranean and Macaronesian halo-nitrophilous semidesert scrub” with several subcategories, which immediately has an ecological meaning for tick research (references are included in the main body of the text of the manuscript).
- Questing (or host-seeking): a behaviour of most hard ticks consisting of waiting on the vegetation to meet a host.
- Voucher specimen: a tick specimen from which a molecular sequence was obtained that is kept in a collection, and freely loaned to researchers who want to examine it. This is mainly carried out to compare the morphology of the voucher specimen with other specimens and to conciliate both morphological and molecular identifications.
